# Paediatric and Adolescent Breast Cancer: A Narrative Review

**DOI:** 10.7759/cureus.48983

**Published:** 2023-11-18

**Authors:** Natalie Hassan, Philip Idaewor, Noreen Rasheed, Abdalla Saad Abdalla Al-Zawi

**Affiliations:** 1 Critical Care, Basildon University Hospital, Basildon, GBR; 2 Histopathology/Cellular Pathology, Mid and South Essex National Health Service (NHS) Foundation Trust, Basildon, GBR; 3 Histopathology/Cellular Pathology, Basildon and Thurrock University Hospital, Basildon, GBR; 4 Radiology, Basildon and Thurrock University Hospital, Basildon, GBR; 5 General and Breast Surgery, Basildon and Thurrock University Hospital, Basildon, GBR; 6 General and Breast Surgery, Anglia Ruskin University, Chelmsford, GBR; 7 General and Breast Surgery, Mid and South Essex National Health Service (NHS) Foundation Trust, Basildon, GBR

**Keywords:** etv6-ntrk3 fusion gene, brca gene mutation, mastectomy, secretory breast carcinoma, paediatric breast cancer

## Abstract

Breast cancer is the most prevalent form of cancer worldwide. Every year, it affects a significant number of women in the UK and is considered one of the leading causes of cancer-related deaths globally. While breast cancer is primarily linked to adult women, its occurrence in children and adolescents is exceedingly rare. This study conducted a narrative review spanning from 1999 to 2023, examining 32 case reports to investigate the characteristics of breast cancer in the paediatric age group. These reports focused on patients under 18 years old who were diagnosed with primary glandular breast cancer, excluding cases originating from other tissues like angiosarcoma, leukaemia, and metastatic cancer. The data analysis encompassed various parameters, including gender, age, histology, receptor status, lymph node involvement, treatment methods, and genetic characteristics. From the published case reports, it was concluded that the most common type of breast cancer affecting children and adolescents is secretory breast carcinoma and predominantly occurs in females. It is typically hormone receptors negative, and the preferred treatment approach involves mastectomy as breast conservation surgery to preserve the developing breast tissue is a real challenge due to limited breast tissue volume in this age group.

## Introduction and background

Breast cancer is the most encountered malignancy globally after the exclusion of non-melanomatous skin neoplasm, with approximately 2.3 million new cases each year, making up 12% of all newly diagnosed cancer cases [1,2].

The observation from the last three decades revealed that its incidence and mortality rates are climbing up as a result of the change in risk factor profiles, improved cancer registry, and implementation of cancer detection projects [1,2]. According to the UK Breast Cancer Trust, there are around 56,000 new breast cancer diagnoses annually in the UK, constituting 15% of all new cancer cases. Furthermore, it ranks as the fifth leading cause of cancer-related deaths worldwide [3,4]. In 2021, Lukasiewicz et al. published an updated review of epidemiology, risk factors, classification, prognostic markers, and current treatment strategies of breast cancer. They have reported that about 80% of breast cancer cases affected women aged 50 and above [1]. Although breast cancer primarily impacts adults, it does occur rarely in children and adolescents, making up less than 0.1% of breast cancer cases and less than 1% of paediatric cancer cases [5]. This paper aims to investigate the characteristics of breast cancer in the paediatric age group through a narrative review of the available literature.

## Review

This narrative review includes 32 case reports which have been retrieved from published literature (Google Scholar and Pubmed) from the year 1999 to the year 2023. Case reports which are included in this narrative review are patients with confirmed primary breast cancer diagnoses who are younger than 18 years old. Breast cancers which have not originated from the breast glandular tissue (angiosarcoma/leukaemia/metastatic cancer) were excluded. The data collected from the case reports included different parameters like gender, age, histology, receptor status, lymph node status, treatment options, and genetic profile (Table 1).

**Table 1:** Epidemiological and biological characteristics of 32 breast cancers diagnosed in paediatric and adolescent age groups. DCIS: Ductal carcinoma in situ, IDC: Invasive ductal carcinoma, ITC: Invasive tubular carcinoma, SBC: Secretory breast carcinoma, PIC: Papillary intracystic carcinoma, ND: Not done

Author	Year	Gender	Age	Type of Cancer	ER Receptor	PR Receptor	HER2 Receptor	Gene Test	Type of Surgery
Longo [6]	1999	F	4	SBC	ND	ND	ND	ND	Mastectomy
Titus [7]	2000	M	9	SBC	-ve	-ve	ND	ND	Mastectomy
Murphy [8]	2000	F	6	SBC	-ve	-ve	ND	ETV6-NTRK3 fusion gene/BRCA 1-2 negative	Mastectomy
Bree [9]	2002	M	17	SBC	ND	ND	ND	ND	Mastectomy
Bond [10]	2004	F	9	SBC	ND	ND	ND	ND	Mastectomy
Buchino [11]	2004	F	9	SBC	ND	ND	ND	ND	Wide Local Excision
Szanto [12]	2004	M	7	SBC	-ve	-ve	ND	BRCA 1-2 negative	Mastectomy
Wadie [13]	2005	M	16	DCIS	+ve	+ve	ND	ND	Mastectomy
Corroppolo [14]	2008	M	15	DCIS	ND	ND	ND	ND	Mastectomy
Syeed [15]	2010	M	14	IDC	+ve	+ve	ND	CAV-1 mutation	Mastectomy
Engelman [16]	2011	F	7	SBC	-ve	-ve	-ve	ND	Mastectomy
Yorozuya [17]	2011	F	9	SBC	+ve	-ve	-ve	ETV6-NTRK3 fusion gene	Mastectomy
Cabello [18]	2012	M	13	SBC	-ve	-ve	-ve	ND	Mastectomy
Hamza [19]	2012	M	11	SBC	ND	ND	ND	ND	Mastectomy
Tadesse [20]	2012	F	7	SBC	+ve	+ve	ND	ND	Mastectomy
Fathi [21]	2013	F	11	SBC	-ve	-ve	-ve	BRCA 1-2 negative	Mastectomy
Sato [22]	2013	F	17	DCIS	+ve	+ve	ND	ND	Mastectomy
Simpson [23]	2013	F	15	ITC	+ve	+ve	-ve	BRCA 1-2 negative	Mastectomy
Ahmed [24]	2014	F	11	IDC	-ve	-ve	-ve	ND	Mastectomy
Kim [25]	2014	F	14	IDC	-ve	-ve	-ve	BRCA 1-2 negative	Wide Local Excision
Wang [26]	2014	F	12	SBC	-ve	-ve	-ve	ND	Breast Conservative Surgery
Soyer [27]	2015	F	6	SBC	-ve	-ve	ND	ND	Mastectomy
Misra [28]	2016	M	8	SBC	+ve	-ve	-ve	ETV6-NTRK3 fusion gene	Mastectomy
Mohamed [29]	2016	M	12	SBC	-ve	-ve	-ve	ND	Mastectomy
Garlick [30]	2017	F	8	SBC	+ve	-ve	-ve	ETV6-NTRK3 fusion gene	Mastectomy
Li [31]	2017	F	7	SBC	-ve	-ve	-ve	No mutation	Mastectomy
Ghilli [32]	2018	M	6	SBC	-ve	-ve	-ve	ETV6-NTRK3 fusion gene; BRCA 1-2 negative	Wide Local Excision
Gohara [33]	2020	F	6	SBC	+ve	-ve	-ve	ND	Excisional Biopsy - Local excision refused by parents not to burden the child
Kluppel [34]	2020	M	14	SBC	-ve	-ve	-ve	ND	Mastectomy
Apodaca-Ramos [35]	2021	F	16	PIC	+ve	+ve	ND	No mutation	Mastectomy
Shi [36]	2021	F	8	SBC	+ve	-ve	-ve	ETV6-NTRK3 fusion gene	Wide Local Excision
Mazellier [37]	2023	F	6	SBC	-ve	-ve	-ve	ETV6-NTRK3 fusion gene	Mastectomy

The age of the cases ranged from 4 to 17 years old with a standard deviation of 3.82. The majority of cases were females (62.5%, N=20) compared to males (37.5%, N=12).

Children and adolescents had different clinical presentations where 84% of them (27 cases) had a lump; of which 89% (24 cases) were painless lumps whilst 11% (three cases) were the lump was associated with pain. Out of the 84% presenting with a lump, 26% (seven cases) of them had associated nipple discharge. Six per cent of the patients (two cases) presented with bilateral gynecomastia. Only one case (3%) presented with ulceration associated with pus and bloody discharge, and 3% (one case) presented with nipple discharge associated with severe pain.

Different investigations were used in each of the cases to evaluate the presenting complaint. Nineteen per cent of the cases (six cases in total) had a mammography while 78% (26 cases) had a breast ultrasound. Sixteen per cent of the cases (five cases in total) had a breast MRI whilst one case (3%) had a CT chest to investigate the breast lump. To stage the cancer, 9% of the cases (three cases) had a CT scan of the chest and abdomen whilst 6% (two cases) had a PET CT and 3% (one case) had a bone scan. A total of 12.5% of the cases (four cases) relied on the USG abdomen and 16% of the cases (five cases) relied on chest radiographs for staging.

**Figure 1:**
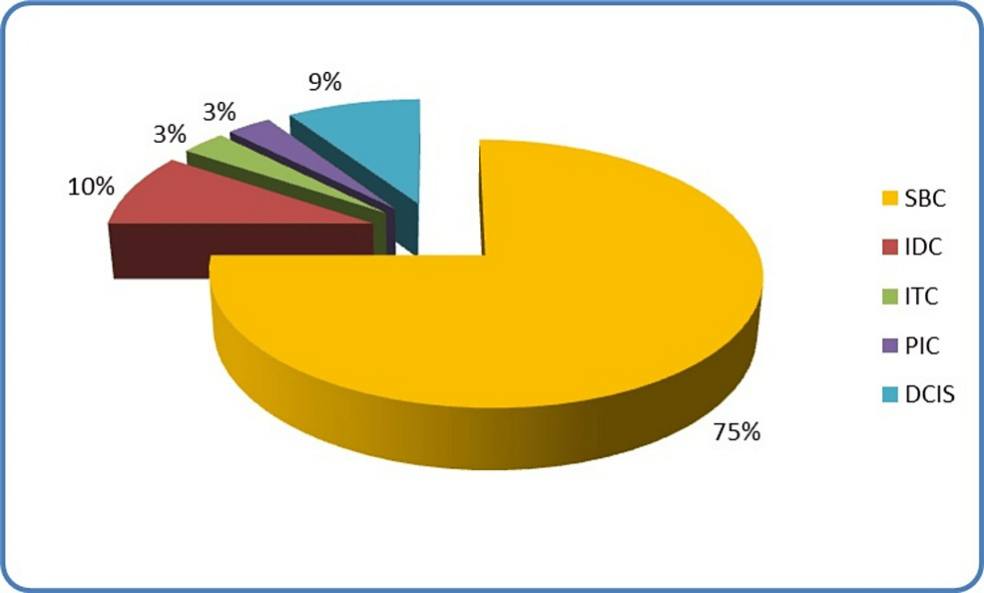
Histological subtypes of breast cancers in 32 cases of paediatric and adolescent patients. SBC: Secretory breast carcinoma, IDC: Invasive ductal carcinoma, ITC: Invasive tubular carcinoma, PIC: Papillary intracystic carcinoma, DCIS: Ductal carcinoma in-situ

The majority of the breast cancers in the cohort were secretory breast carcinoma (SBC), which accounted for 75% of the cases. Besides, 9% of the cases were ductal carcinoma in situ (DCIS), 10% were invasive ductal carcinoma, 3% were invasive tubular carcinoma, and 3% were papillary intracystic carcinoma (Figure 1).

**Figure 2:**
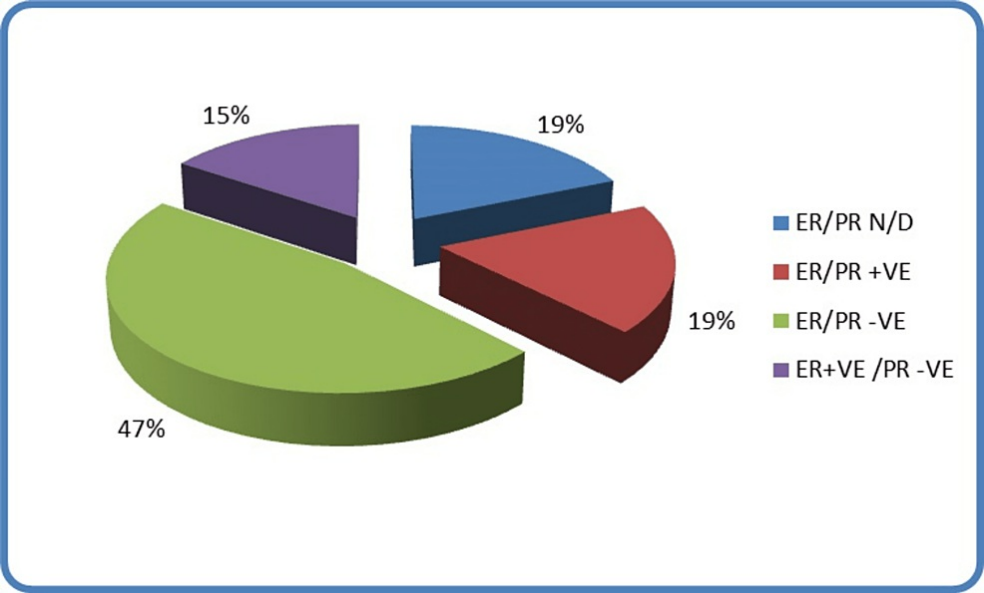
Hormonal profile of paediatric and adolescent breast cancer ER: Estrogen receptors, PR: Progesterone receptors, N/D: Not done

Hormone receptor status is a crucial element in breast cancer diagnosis and management. Unfortunately, six of the 32 cases didn’t report the hormone receptor status which accounts for 19% of the cases. However, the majority of the tested cases (47% of the cohort), were hormone receptor-negative. Nineteen per cent of the cases were both oestrogen and progesterone hormone receptor-positive whilst the remaining 15% were oestrogen receptor-positive but progesterone receptor-negative (Figure 2).

As for the human epidermal growth factor receptor 2 (HER-2) receptor status, 53% of the cases were negative whilst the remaining 47% of the cases had no reported HER-2 status. None of the tested cases were reported to have a HER-2 positive.

Further, 56% of the cases didn’t have any genetic testing. Nineteen per cent of the cases had genetic testing without any mutation detected. Six per cent of the cases were BReast CAncer gene (BRCA) 1/2 negative, but had ETV6-NTRK3 fusion gene. Sixteen per cent of the cases had the ETV6-NTRK3 fusion gene but were not tested for the BRCA1/2 gene. Three per cent (one case) had a CAV-1 mutation. The majority of patients (81%) had mastectomy whilst 15.6% of them had wide local excision. One of the patients was offered wide local excision after the excisional biopsy, however, her parents refused to proceed with the surgery in order not to burden their child. Breast cancer is regarded as the most common female malignancy and one of the most important causes of cancer-related mortality among them, about 80% of breast cancer patients are aged >50 years. Breast malignancy in children and adolescents is exceptionally rare, resulting in very limited published literature consisting mostly of case reports. Nevertheless, it does occur, and it's crucial for patients and parents to be aware of this potential risk. While it can affect males, it is more commonly seen in females [24]. The predominant form of breast cancer affecting children and adolescents is SBC, originally termed "juvenile breast cancer" by McDivitt and Stewart in 1966 [26,37]. However, in the 1980s, Tavasolli and Norris identified this type of breast cancer in adults, and thus it was renamed "Secretory Breast Carcinoma" based on its histological characteristics [26,27,38]. These histological features include abundant secretions of sulfated mucopolysaccharides and mucin intracellularly and extracellularly, along with granular eosinophilic cytoplasm [18,32,34,38]. Typically, children and teenagers with SBC present with a slow-growing, painless breast lump [34]. Nipple discharge and retraction may occur but are relatively uncommon [8,35]. Due to its rarity and the lack of pain in its presentation, the diagnosis of breast cancer can often be delayed [19,24].

Because breast lumps are so rare in this age group, there is no standardized approach for investigating them. While mammography is the preferred diagnostic tool for adults, its use in children is limited due to poor image quality and radiation exposure hazards [8]. The available literature suggests that breast ultrasonography is the primary method for investigating breast lumps in children and adolescents due to accessibility, diagnostic specificity and avoiding unnecessary radiation exposure [18,25,31]. Often, on ultrasonography the lesions are seen as round or oval abnormality, with circumscribed or partially micro-lobulated margins and in relation to the surrounding fatty tissue, they are hypoechoic with non-homogeneous internal echoes [39,25]. When ultrasound imaging is inconclusive, breast MRI (magnetic resonance imaging) may be used, although its use, generally, is limited in the paediatric population [24,31,37]. Breast MRI is advantageous for identifying deeper structures and vascular and lymphatic malformations, but it was demonstrated to have a modest false-negative rate in a study presented by Teifke et al. in 2020 [40]. Fine needle aspiration (FNA) or core biopsy in indeterminate/suspicious is commonly used in adults, but its use in children and adolescents is limited (Cabello, 2012), it is a quick and minimally invasive procedure, making it the preferred choice over excisional biopsy [9,24]. Excisional biopsy, on the other hand, might negatively impact the developing breast bud, leading to deformity and breast asymmetry [18,19,24]. The current evidence supports that, the gold standard for evaluating breast lumps in women under 30 involves a triple assessment approach, comprising clinical assessment, ultrasound examination, and imaging-guided core needle biopsy [39]. Moreover, in children and adolescents, SBC is typically negative for estrogen and progesterone receptors (ER/PR) and HER-2. Although there have been case reports with ER-positive tumours, these are associated with a poorer prognosis [24,34]. More than 90% of SBC cases result from a genetic mutation characterized by a translocation between chromosomes 12 and 15, leading to the expression of the ETV6-NTRK3 fusion gene. This gene produces an active tyrosine kinase that plays a crucial role in activating pathways responsible for breast cell proliferation and survival [30,32,37]. This translocation is not associated with other forms of breast cancer but has been linked to congenital fibrosarcoma, mesoblastic nephroma, acute myeloid leukaemia, and secretory carcinoma of the skin [32,34]. The treatment of SBC in children and adolescents remains a subject of debate due to the scarcity of evidence in the literature. Ahmed et al. in 2014 suggest that it should be treated with wide local excision whenever possible. Efforts should be made to preserve the developing breast tissue to avoid impairing normal breast development, but in most cases, a mastectomy is necessary as the ultimate treatment. Radiotherapy should be avoided in all circumstances due to its potential side effects, including lung fibrosis, rib damage, asymmetry of the rib cage, and long-term effects on surrounding skin and breast tissue, as well as an increased risk of secondary malignancies due to the long-life expectancy of these patients [18,24,25,30]. Breast cancer originates from the glandular epithelial tissue and has various subtypes, which differ in their biological characteristics, treatment strategy, metastasis tendency, prognosis and survival rates. Breast cancer survival depends on the disease stage at diagnosis and molecular subtype. Fortunately, SBC has a slow-growing pattern as well as characterized by extremely rare distant metastases [12,19,21], the tendency for late local recurrence and prolonged survival even with lymph node metastases. The mortality due to metastatic secretory carcinoma is extremely rare [37], in general, patients with SBC have a favourable prognosis with a five-year overall survival of 87% [41].

## Conclusions

In summary, breast cancer is exceptionally uncommon among the paediatric age group, representing a minute proportion of both breast cancer and paediatric malignancies. The prevailing type of breast cancer in children and adolescents is secretory breast cancer, primarily affecting females, yet it holds a favourable prognosis when identified in its early stages. Further research work is needed to fully understand the behaviour of paediatric breast cancer and genomic profiling is recommended in every case to improve case management.
